# Direct observation of narrow mid-infrared plasmon linewidths of single metal oxide nanocrystals

**DOI:** 10.1038/ncomms11583

**Published:** 2016-05-13

**Authors:** Robert W. Johns, Hans A. Bechtel, Evan L. Runnerstrom, Ankit Agrawal, Sebastien D. Lounis, Delia J. Milliron

**Affiliations:** 1 Department of Chemistry, University of California, Berkeley, California 94720, USA; 2 McKetta Department of Chemical Engineering, The University of Texas at Austin, 200 East Dean Keeton Street, Austin, Texas 78712, USA; 3 Advanced Light Source, Lawrence Berkeley National Laboratory, Berkeley, California 94720, USA; 4 Department of Materials Science and Engineering, University of California, Berkeley, California 94720, USA; 5 Graduate Program in Applied Science and Technology, University of California, Berkeley, California 94720, USA

## Abstract

Infrared-responsive doped metal oxide nanocrystals are an emerging class of plasmonic materials whose localized surface plasmon resonances (LSPR) can be resonant with molecular vibrations. This presents a distinctive opportunity to manipulate light–matter interactions to redirect chemical or spectroscopic outcomes through the strong local electric fields they generate. Here we report a technique for measuring single nanocrystal absorption spectra of doped metal oxide nanocrystals, revealing significant spectral inhomogeneity in their mid-infrared LSPRs. Our analysis suggests dopant incorporation is heterogeneous beyond expectation based on a statistical distribution of dopants. The broad ensemble linewidths typically observed in these materials result primarily from sample heterogeneity and not from strong electronic damping associated with lossy plasmonic materials. In fact, single nanocrystal spectra reveal linewidths as narrow as 600 cm^−1^ in aluminium-doped zinc oxide, a value less than half the ensemble linewidth and markedly less than homogeneous linewidths of gold nanospheres.

## Abstract (web summary)

Establishing the cause of inhomogeneous broadening would help to produce narrow ensemble localized surface plasmon resonance peaks, favourable for sensing applications. Here, Johns *et al*. use near field optics for enhancing signal contrast, enabling the measurement of mid-infrared spectra of single nanocrystals.

## Graphical abstract

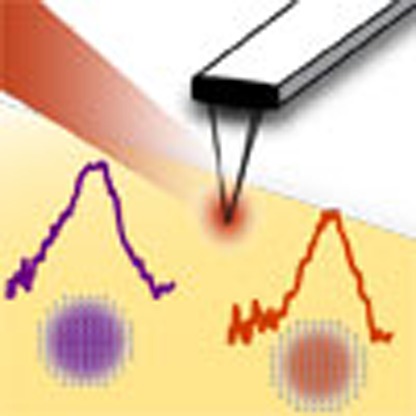

Plasmonic materials with infrared resonances can concentrate far-field radiation^1^,^2^,^3^,^4^ at energies where biological environments are transparent, where molecular vibrations are resonant, and where solar energy is abundant enabling applications in sensing and energy harvesting^5^,^6^,^7^. Hence, these stable, few-nanometre colloidal particles are promising for enhancing light–matter interactions and spectroscopies in this important spectral region, where detection of signals is often most difficult. The development of such applications hinges on maximizing the electric field enhancement, which varies inversely with the strength of damping of the free electron oscillation^8^. Spectral linewidth measurements provide a convenient handle to study damping because damping is also proportional to the homogenous linewidth of localized surface plasmon resonances (LSPR) peaks for spherical particles^8^,^9^. However, in metal oxide nanocrystals intrinsic linewidths have so far been obscured by undetermined heterogeneous contributions to peak widths obtained in ensemble measurements^10^. Also, sharp ensemble peak widths are favourable for sensing applications, since shifts of broad peaks can be more challenging to detect and quantify^9^,^11^. Establishing the extent and cause of inhomogeneous broadening would inform future efforts to produce narrow ensemble LSPR peaks.

Room temperature LSPR homogenous linewidths of metal nanoparticles are typically obtained through detection of single nanoparticle extinction or scattering spectra with methods like dark field scattering^12^, interferometric detection,^13^ and spatial modulation spectroscopy^14^. These techniques rely on single particles interacting with enough of the focused light to create detectable contrast, which has been successful at visible wavelengths used to probe metal nanoparticles^15^,^16^. However, longer wavelength infrared light cannot be focused to as small a diffraction-limited spot, making such far-field methods infeasible for probing LSPR spectra of our ∼20 nm metal oxide nanocrystals. While the volume ratio of a 20 nm particle to a diffraction-limited spot of 500 nm wavelength visible light is about 1.4 × 10^−3^, this ratio drops to 1.4 × 10^−6^ for 5 μm wavelength mid-infrared light, so the signal would be lost in an overwhelming background. Therefore, an alternative approach is needed.

We found that near-field optics were ideal for enhancing signal contrast, enabling us to measure mid-infrared LSPR spectra of single nanocrystals. Specifically, we used synchrotron infrared nano-spectroscopy (SINS)^17^ to directly record the LSPR spectra of individual aluminium-doped zinc oxide (AZO) and tin-doped indium oxide (ITO) nanocrystals. The observed variations in spectral characteristics, including resonance energy, peak shape and linewidth, were statistically evaluated for correlations with nanocrystal size and compared with simulated LSPR spectra to determine the most significant sources of spectral heterogeneity. Our analysis indicates that doping variations are a dominant factor influencing spectral heterogeneity, so efforts to reduce heterogeneous broadening should not only target uniform nanocrystal shapes as would be true for metal nanoparticles, but also uniform dopant distributions. Nonetheless, the narrowest single nanocrystal linewidths measured (600 cm^−1^) are less than half the linewidths of the corresponding ensembles and narrower than LSPR peaks of gold nanoparticles, indicating low intrinsic damping and suggesting that plasmonic oxide nanocrystals hold great potential to enable a wide range of applications by strongly concentrating infrared light at nanometre length scales.

## Results:

### Evaluation of nanocrystal heterogeneity with electron microscopy

**Figure 1:**
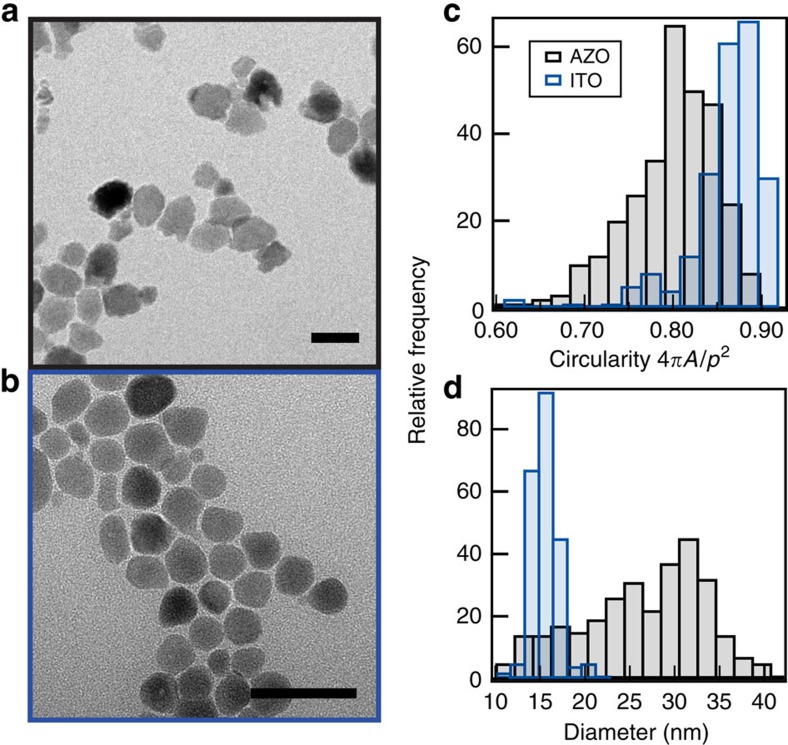
AZO and ITO nanocrystals structural heterogeneity. Transmission electron micrographs of (**a**) AZO with an average diameter of ∼30 nm and (**b**) ITO with an average diameter of ∼15 nm. Scale bars, 20 nm. Histograms of (**c**) shape and (**d**) size distributions indicate that the ITO nanocrystals are smaller, more spherical, and more uniform in shape and size than the AZO nanocrystals.

**Figure 2:**
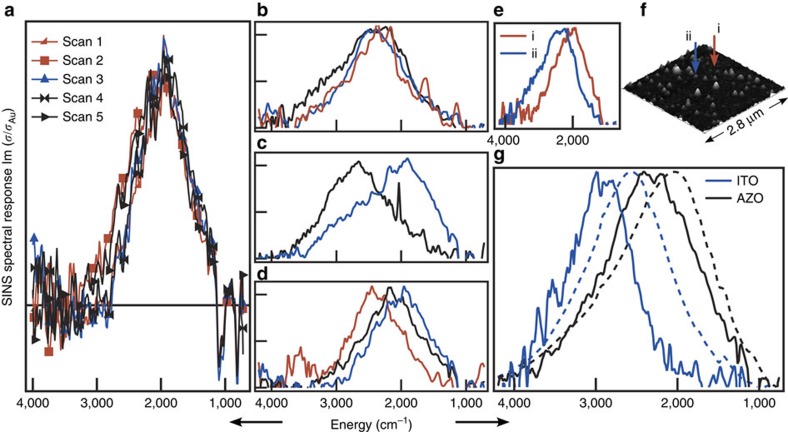
Heterogeneity observed by single nanocrystal spectroscopy of AZO. (**a**) Repeated scans of an individual nanocrystal reproduce the same spectrum. A horizontal line at zero is included. Variations among nanocrystals were observed in (**b**) linewidth, (**c**) peak asymmetry and (**d**) peak energy. The eight spectra shown in **b**–**d** are each an average of several scans of an individual nanocrystal. Single nanocrystal spectra (**e**) are correlated with topographic information from AFM (**f**). (**g**) The sums of all collected single nanocrystal spectra (solid lines) form pseudo-ensembles for comparison to ensemble spectra measured by conventional transmission FTIR of thin films on KBr substrates (dashed lines) for both AZO and ITO.

We compared two representative colloidal doped metal oxide nanocrystal materials, 1% doped ITO and 1.5% doped AZO, with differing size and shape polydispersity (Fig. 1, Supplementary Methods). Besides shape and size heterogeneity, which are present for all colloidal nanoparticles, doping introduces additional sources of spectral heterogeneity; variations in dopant concentration, the spatial distribution of dopants^18^,^19^ and the ratio of dopants to free electrons are all possible. Though dopant-related heterogeneity is challenging to directly assess, we evaluated size and shape heterogeneity by analysis of transmission electron microscopy (TEM) images (Fig. 1). The shape distribution was quantified using a circularity parameter (*C*=4*πA/p*^2^) typical for description of spheroidal particles via their two-dimensional (2D) projections observed in electron micrographs, where *A* is the area and *p* is the perimeter of the nanoparticle. On average, the AZO nanocrystals are less spherical and more polydisperse in their circularity, although no distinct elongation or particular shape was expressed. In addition, the AZO nanocrystals averaged about eight times larger in volume than ITO nanocrystals and had a broader size distribution. Non-spherical particles^19^ can exhibit LSPR shifts in both energy and linewidth depending on the orientation of light polarization versus the particle orientation. In addition, particles that exceed a certain size threshold (*λ*_LSPR_/20) can start to experience size-induced energy shifts. *A priori*, these size and shape inhomogeneities might be assumed to explain the broader ensemble linewidth of AZO compared with ITO (Fig. 2g). For nanocrystals with mid-infrared LSPR, however, size-dependent shifts would only be expected for particle diameters over 100 nm, and shape effects generally manifest only for particles with significant faceting or elongation along a particular dimension. Alternatively, the intrinsic damping might be more substantial in AZO than ITO, in which case broad linewidths should also be observed for individual AZO nanocrystals. So, we sought to directly measure homogeneous linewidths and determine the significant contributions to heterogeneous broadening by using SINS to collect extinction spectra of single nanocrystals.

### Single nanocrystal optical characterization

The combination of a bright, broadband infrared source with high spatial resolution makes SINS ideal for these experiments. Although supercontinuum laser-based sources are generally brighter, the spectral bandwidth of these is limited to ∼700 cm^−1^ (ref. 20), which is insufficient to record the full width of an LSPR peak. With SINS, a spectral range of 4,000 cm^−1^ is available in a single scan, so it is possible to collect an entire spectrum without tuning the source, thereby reducing systematic errors in the measurement. To probe a sub-diffraction-limited region, synchrotron infrared light is focused onto a conductive atomic force microscopy (AFM) tip, localizing the electric field in a small region around the tip apex (<25 nm). The ligand-capped nanocrystals were deposited on a gold substrate, which improves signal-to-noise, effectively creating a near-field cavity to enhance the interaction of infrared light with the nanocrystal under interrogation. Topographic data were collected concurrently, enabling us to compare nanocrystal size and spectroscopic features (Fig. 2e,f). In addition, fiducial markers on our substrates facilitated post-spectral scanning electron microscopy (SEM) analysis to verify that single nanocrystals were measured (Supplementary Note 1, Supplementary Figs 1 and 2). Backgrounds were collected from nearby regions of the gold substrate. By locking in on the second harmonic of the AFM tapping frequency, the near-field signal could be isolated from the far-field background, improving sensitivity and signal-to-noise ratio. Single particle spectra are reported as SINS spectral response, which is the imaginary component of the complex scattering function *σ*(*ω*), yielding a spectrum that is analogous to an absorption measurement, as described previously^20^. These scattering functions are the ratio of the material response to that of the background (signal processing is discussed further in Supplementary Note 2). Nanocrystals were selected for analysis by choosing well-isolated individuals rather than by size to minimize selection bias. Although the lateral dimensions of the nanocrystals were not well-resolved by AFM, the AFM-derived height distribution of the analysed nanocrystals correlates well with the size distribution observed by TEM (Supplementary Note 3, Supplementary Fig. 3).

Notably, these nanocrystals are the smallest objects from which SINS IR spectra have been collected^21^. It is important to evaluate this new technique to be sure that the observations reflect material characteristics and are not artifacts of the experimental method. The nanocrystal LSPR is probed utilizing the near-field hot spot created between the tip and substrate, so one concern is that tip–particle interactions might induce spectral changes, which would vary from one measurement to the next. Indeed, it has been reported that tip-to-particle positioning impacts the intensity of light scattered by silver nanostructures^22^. For each (AZO and ITO) sample, all single nanocrystal spectra collected were summed to generate pseudo-ensemble spectra for comparison to ensemble spectra collected by conventional far-field Fourier transform infrared (FTIR) transmission spectroscopy of thin films (Fig. 2g). The ensemble linewidths are reproduced well in the pseudo-ensemble spectra, while the far-field peaks are shifted to lower energy compared with their SINS-derived counterparts, which is a direct result of the particles interacting with a metal substrate in SINS rather than a dielectric substrate for FTIR^23^,^24^ (Supplementary Table 1, Supplementary Fig. 4, Supplementary Note 4). We note that LSPR spectra shift systematically depending on the measurement geometry and that thicker films can exhibit spectral broadening, which can account for differences between the pseudo-ensembles, ensemble spectra measured in reflectance, and those measured in transmittance (Supplementary Figs 4 and 5). The general consistency of the ensemble and pseudo-ensemble lineshapes, however, lends confidence that SINS spectra faithfully reflect optical characteristics of each nanocrystal and are not significantly distorted by near-field coupling to the tip or substrate.

Furthermore, repeated scans on a single nanocrystal were highly reproducible. A series of spectra were collected, moving the tip away from the nanocrystal between each to collect a new background scan from a distinct area of the gold surface. The resulting LSPR spectra were consistent (Fig. 2a). The negative peaks apparent at 802; 1,095; and 1,265 cm^−1^ are ascribed to residual poly(dimethylsiloxane) adsorbed on the AFM tip^25^ (discussed further in Supplementary Note 2).

**Figure 3:**
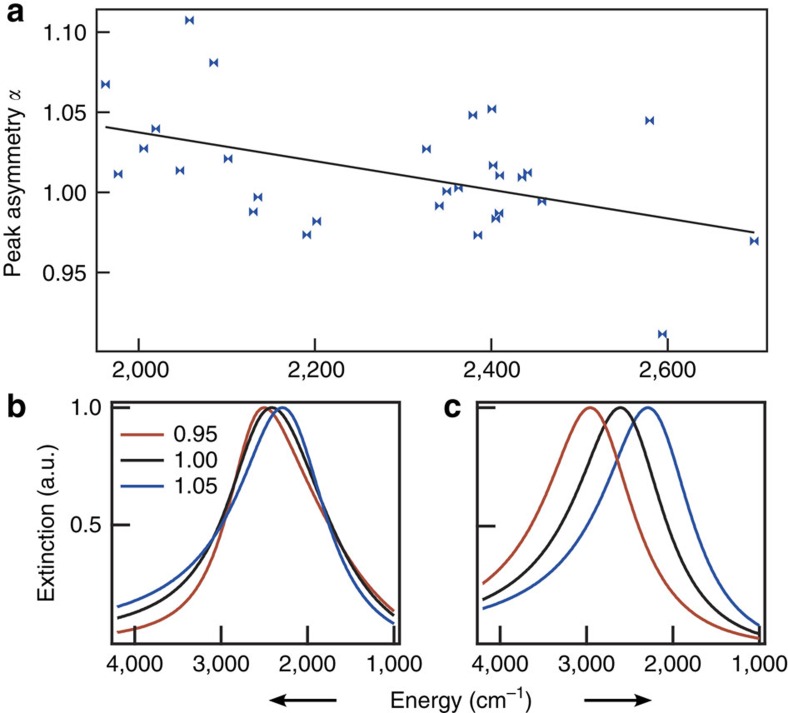
Sources of heterogeneity in single nanocrystal LSPR spectra. (**a**) Experimental observation of correlation between peak energy and peak asymmetry. (**b**) Simulations of peak shapes resulting from variations in the strength of frequency-dependent damping can account for heterogeneity in peak asymmetry. The *α* value of each peak is shown. (**c**) Simulated spectra over a range of electron concentrations account for variations in peak energy.

Comparing the LSPR spectra of individual AZO nanocrystals, variations in peak linewidth, peak shape, and energy are apparent (Fig. 2b–d, Supplementary Table 1). In contrast to previous reports, where size variations were proposed to account for peak energy variations in spectra of single gold nanoparticles^8^,^16^, we found no correlation between nanocrystal size (as determined by AFM height) and peak energy (Supplementary Note 5, Supplementary Figs 6 and 7). This is consistent with Mie theory as these particles are substantially smaller than the quasi-static limit for mid-infrared LSPR. For the AZO nanocrystals, the s.d. of peak energies about the mean of 2,285 is 201 cm^−1^, or about 9%, which could instead be caused by variations in electron concentration between individual nanocrystals. We tested this hypothesis by using Mie theory to predict the deviations in electron concentration needed to reproduce the observed variations in LSPR peak energy. In the simulations (Fig. 3c, Supplementary Note 6, Supplementary Table 2), a ±25% change in electron concentration can approximately account for the full range of spectral heterogeneity found in our experimental results. This distribution in the electron concentrations among individual nanocrystals may reflect variations in dopant (Al^3+^) concentration or activation of dopants to yield free electrons^26^. Single particle energy dispersive spectroscopy was performed on several nanocrystals in the TEM to evaluate variations in Al^3+^ concentration. A range of 1.3–2.0% was observed, which is consistent with the distribution of LSPR energies measured by SINS. The range of dopant concentrations that a statistically random distribution of dopants would yield is an order of magnitude less than that suggested by SINS and directly observed by EDS (Supplementary Note 7).

The shape of the LSPR peak also varies between individual nanocrystals (Fig. 2c), which we describe by defining a peak asymmetry parameter (*α*) as the ratio of the average of the two half-maximum energy values to the peak energy. A value of *α*>1 reflects asymmetric broadening towards high frequency, while *α*<1 peaks are broadened towards low frequency and *α*=1 describes a symmetric peak. The peak asymmetry parameter has a statistically significant (*P*<0.01) correlation with peak energy (Fig. 3a and Supplementary Fig. 8), suggesting that these two sources of heterogeneity have a common origin. In doped metal oxides, scattering from ionized impurities is known to give rise to frequency-dependent damping^19^,^26^ that causes asymmetric broadening of the LSPR peak towards lower frequencies (*α*<1)^1^. Hence, higher dopant concentration in a given nanocrystal could be expected to increase the peak energy via increase in electron concentration and simultaneously decrease in the asymmetry parameter *α* due to ionized impurity scattering by the same dopants, consistent with our experimental observation. A cross-over from *α*>1 to *α*<1 with increasing dopant-induced, frequency-dependent damping is also apparent in simulated spectra (Fig. 3b and Supplementary Table 3). The peak energy also shifts slightly to higher energy as a direct result of variable damping, though changes in electron concentration are needed to account for the larger range of the experimentally observed peak energies (Fig. 3c). We note that various peak shapes can also arise from multiple spatial modes of LSPR excitation in particles with complex, non-spherical shapes^27^, though we would not expect this source of heterogeneity in *α* to correlate with peak energy. Hence, two important sources of heterogeneous broadening in metal oxide LSPR spectra—peak energy and peak shape variations—derive significantly from doping heterogeneity. These factors are absent in metal nanoparticle ensembles, suggesting that ensemble measurements may significantly overestimate damping of doped metal oxide LSPRs.

## Discussion

**Figure 4:**
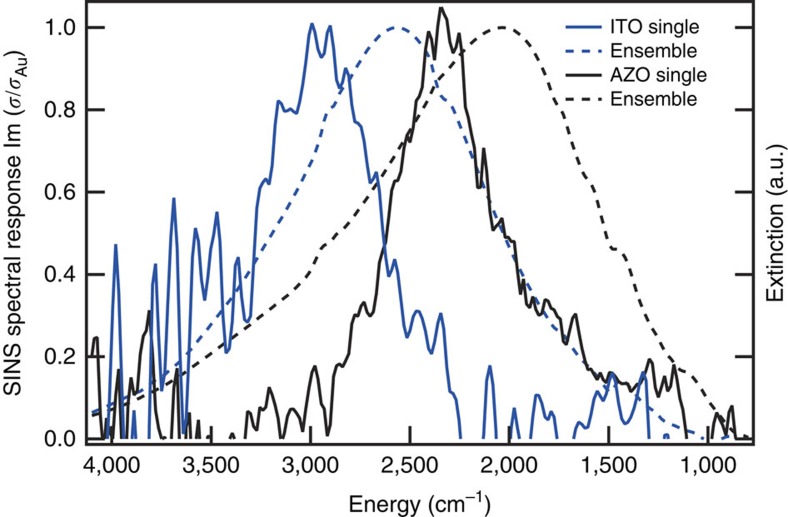
Homogeneous linewidths of metal oxide nanocrystal LSPRs. The spectra of the single ITO (blue) and AZO (black) nanocrystals with the narrowest observed linewidths (solid lines) are compared with the associated ensemble spectra (dashed lines).

Indeed, the narrowest single nanocrystal LSPR linewidths we observed are about half of the ensemble linewidths (Fig. 4) and are even narrower than those found by measuring single metal nanoparticles at room temperature. Linewidths of single gold nanospheres have been reported to be as low as 970 cm^−1^ (120 meV)^8^, while we measured linewidths of 600 cm^−1^ in individual ITO and AZO nanocrystals. Considering that coupling to the near-field would be expected to broaden the LSPR peaks, if anything, these narrow peaks set an upper bound on the homogeneous broadening and predict that dephasing times for metal oxide LSPR excitations are 18 fs at the shortest (Supplementary Note 8). The corresponding *Q* factors (the ratio of the peak energy to the linewidth) are 4.5 for ITO and 3.9 for AZO. *Q* factors are used to describe how energy is damped out of harmonic oscillators, which tracks with the local fields created by LSPRs. Such low intrinsic damping suggests these materials are promising for applications involving field enhancement. However, the single nanocrystal spectra exhibit a wide range of linewidths, with the s.d. of full-width-at-half-maximum values for AZO and ITO being 238 cm^−1^ and 171 cm^−1^, respectively (Fig. 2b and Supplementary Fig. 9). Recalling the greater shape heterogeneity in our AZO nanocrystals (Fig. 1), we expect that significant broadening found in many of the single nanocrystal spectra can result from multiple spatial modes found in nanocrystals with complex shapes. Importantly, this source of broadening does not imply plasmon damping or diminish the prospects for developing field enhancement applications of these nanocrystals. (Contributions to linewidths and damping are discussed further in Supplementary Note 9).

Through the use of synchrotron-based single nanocrystal spectroscopy, ensemble LSPR spectra of metal oxide nanocrystals were found to be substantially broadened by dopant-related heterogeneity. Significant variations in dopant concentration among nanocrystals may result from the kinetic mechanisms used for doped nanocrystal synthesis^28^,^29^ since a Poisson distribution of dopants would produce far less variation in LSPR peak energies. For sensing applications, the ensemble linewidth should be as narrow as possible; our data suggest that improving shape and doping uniformity are important goals to minimize heterogeneous broadening. Regarding field enhancement, the narrow linewidths we measured for single nanocrystals suggest that intrinsic damping of metal oxide LSPRs may be less than that in metal nanoparticles, opening the door to compelling applications. Considering these nanocrystals respond resonantly to infrared light while maintaining small dimensions, there is a distinctive opportunity to couple their LSPRs with molecular vibrations to enhance spectroscopic signals or to influence pathways of surface-bound chemical reactions. Furthermore, because these LSPRs can be tuned dynamically by chemical^30^,^31^,^32^ or electrochemical charging^33^, such coupling could be modulated to create a new class of active systems, manipulating light–matter interactions to redirect chemical or spectroscopic outcomes.

## Methods

### Preparation of substrate-bound isolated nanocrystals

AZO and ITO nanocrystals were synthesized using colloidal chemistry, as discussed fully in the Supplementary Information. Nanocrystals were washed and then dispersed in 50/50 vol%/vol% hexane/octane, these solutions were used to spin coat ligand-bound nanocrystals onto clean substrates of gold and KBr. For SINS measurements, having the highest percentage of isolated nanocrystals possible was crucial so low concentrations were used to ensure good isolation and spacing (Supplementary Fig. 2). For ensemble measurements, these films did not give adequate signal-to-noise so higher concentration solutions were used to create films of close packed nanocrystals in a single monolayer with roughly 50% coverage of the substrate surface, based on SEM.

### TEM analysis

TEM images were collected using a JEOL 2010F operating at 200 kV accelerating voltage. Image analysis of both diameter and circularity was performed using the Analyse Particles function in ImageJ64 using the triangle threshold function. Diameters were calculated from 2D projection areas of particles by treating the particles as spherical.

### Ensemble FTIR measurements

Ensemble measurements were collected on a Bruker Vertex 70 FTIR. Measurements performed on KBr substrates were collected in standard transmission geometry from nanocrystals spin coated onto the surface of 1-mm-thick KBr windows. Due to the low transmission of infrared light through gold, ensemble measurements on gold substrates were collected in the reflection geometry. A polarizer optic was used to select for p-polarization versus the substrate and light was directed onto the sample at 80° from normal. This supported greater signal making it possible to use thinner films of nanocrystals.

### Correlated SEM/AFM

Substrates were prepared by etching 100 nm pitch 7 μm squares into 300 nm of SiO_2_ grown onto a Si wafer. After etching, the substrate was coated by evaporating 5 nm of Cr as a sticking layer and then 50 nm of Au. These squares were discernable optically to determine where the AFM scans should be collected (and provided height contrast for the AFM) and could be seen using a secondary electron detector in Zeiss Gemini Ultra-55 analytical field emission SEM to find the same region. Using these marks to ensure collections where made in the same area, AFM images and SEM images were reconciled against one another to determine which nanocrystal was being measured in the SINS technique (depicted in Supplementary Fig. 2).

## Additional information

**How to cite this article:** Johns, R. W. *et al*. Direct observation of narrow mid-infrared plasmon linewidths of single metal oxide nanocrystals. *Nat. Commun.* 7:11583 doi: 10.1038/ncomms11583 (2016).

## Supplementary Material

Supplementary Information Supplementary Figures 1-9, Supplementary Tables 1-3, Supplementary Notes 1-9, Supplementary Methods and Supplementary References.

## References

[b1] Lounis S. D., Runnerstrom E. L., Llordés A. & Milliron D. J. Defect chemistry and plasmon physics of colloidal metal oxide nanocrystals. J. Phys. Chem. Lett. 5, 1564–1574 (2014). 26270097 10.1021/jz500440e

[b2] Buonsanti R., Llordes A., Aloni S., Helms B. A. & Milliron D. J. Tunable infrared absorption and visible transparency of colloidal aluminium-doped zinc oxide nanocrystals. Nano Lett. 11, 4706–4710 (2011). 21970407 10.1021/nl203030f

[b3] Kanehara M., Koike H., Yoshinaga T. & Teranishi T. Indium tin oxide nanoparticles with compositionally tunable surface plasmon resonance frequencies in the near-IR region. J. Am. Chem. Soc. 131, 17736–17737 (2009). 19921844 10.1021/ja9064415

[b4] Ye X., Fei J., Diroll B. T., Paik T. & Murray C. B. Expanding the spectral tunability of plasmonic resonances in doped metal-oxide nanocrystals through cooperative cation–anion codoping. J. Am. Chem. Soc. 136, 11680–11686 (2014). 25066599 10.1021/ja5039903

[b5] Anger P., Bharadwaj P. & Novotny L. Enhancement and quenching of single-molecule fluorescence. Phys. Rev. Lett. 96, 113002 (2006). 16605818 10.1103/PhysRevLett.96.113002

[b6] Kinkhabwala A. . Large single-molecule fluorescence enhancements produced by a bowtie nanoantenna. Nat. Photon. 3, 654–657 (2009).

[b7] Li Y. . Graphene plasmon enhanced vibrational sensing of surface-adsorbed layers. Nano Lett. 14, 1573–1577 (2014). 24528250 10.1021/nl404824w

[b8] Klar T. . Surface-plasmon resonances in single metallic nanoparticles. Phys. Rev. Lett. 80, 4249–4252 (1998).

[b9] Willets K. A. & Van Duyne R. P. Localized surface plasmon resonance spectroscopy and sensing. Annu. Rev. Phys. Chem. 58, 267–297 (2007). 17067281 10.1146/annurev.physchem.58.032806.104607

[b10] Mendelsberg R. J., Garcia G., Li H., Manna L. & Milliron D. J. Understanding the plasmon resonance in ensembles of degenerately doped semiconductor nanocrystals. J. Phys. Chem. C 116, 12226–12231 (2012).

[b11] Halas N. J., Lal S., Chang W. S., Link S. & Nordlander P. Plasmons in strongly coupled metallic nanostructures. Chem. Rev. 111, 3913–3961 (2011). 21542636 10.1021/cr200061k

[b12] Sönnichsen C. . Drastic reduction of plasmon damping in gold nanorods. Phys. Rev. Lett. 88, 077402 (2002). 11863939 10.1103/PhysRevLett.88.077402

[b13] Lindfors K., Kalkbrenner T., Stoller P. & Sandoghdar V. Detection and spectroscopy of gold nanoparticles using supercontinuum white light confocal microscopy. Phys. Rev. Lett. 93, 037401 (2004). 15323866 10.1103/PhysRevLett.93.037401

[b14] Muskens O. L. . Single metal nanoparticle absorption spectroscopy and optical characterization. Appl. Phys. Lett. 88, 063109 (2006).

[b15] Novo C. . Contributions from radiation damping and surface scattering to the linewidth of the longitudinal plasmon band of gold nanorods: a single particle study. Phys. Chem. Chem. Phys. 8, 3540–3546 (2006). 16871343 10.1039/b604856k

[b16] Olson J. . Optical characterization of single plasmonic nanoparticles. Chem. Soc. Rev. 44, 40–57 (2015). 24979351 10.1039/c4cs00131a PMC4641313

[b17] Bechtel H. A., Muller E. A., Olmon R. L., Martin M. C. & Raschke M. B. Ultrabroadband infrared nanospectroscopic imaging. Proc. Natl Acad. Sci. USA 111, 7191–7196 (2014). 24803431 10.1073/pnas.1400502111 PMC4034206

[b18] Lounis S. D., Runnerstrom E. L., Bergerud A., Nordlund D. & Milliron D. J. Influence of dopant distribution on the plasmonic properties of indium tin oxide nanocrystals. J. Am. Chem. Soc. 136, 7110–7116 (2014). 24786283 10.1021/ja502541z

[b19] Goings J. J. . Theoretical characterization of conduction-band electrons in photodoped and aluminium-doped zinc oxide (AZO) quantum dots. J. Phys. Chem. C 118, 26584–26590 (2014).

[b20] Huth F. . Nano-FTIR absorption spectroscopy of molecular fingerprints at 20 nm spatial resolution. Nano Lett. 12, 3973–3978 (2012). 22703339 10.1021/nl301159v

[b21] Amenabar I. . Structural analysis and mapping of individual protein complexes by infrared nanospectroscopy. Nat. Commun. 4, 2890 (2013). 24301518 10.1038/ncomms3890 PMC3863900

[b22] Rang M. . Optical near-field mapping of plasmonic nanoprisms. Nano Lett. 8, 3357–3363 (2008). 18788789 10.1021/nl801808b

[b23] Maurer T., Adam P. M. & Lévêque G. Coupling between plasmonic films and nanostructures: from basics to applications. Nanophotonics 4, 363–382 (2015).

[b24] Malerba M. . 3D vertical nanostructures for enhanced infrared plasmonics. Sci. Rep. 5, 16436 (2015). 26552340 10.1038/srep16436 PMC4639734

[b25] Lo Y. S. . Organic and inorganic contamination on commercial AFM cantilevers. Langmuir 15, 6522–6526 (1999).

[b26] Jin Z. C., Hamberg I. & Granqvist C. G. Optical properties of sputter-deposited ZnO:Al thin films. J. Appl. Phys. 64, 5117–5131 (1988).

[b27] Agrawal A., Kriegel I. & Milliron D. J. Shape-dependent field enhancement and plasmon resonance of oxide nanocrystals. J. Phys. Chem. C 119, 6227–6238 (2015).

[b28] Erwin S. C. . Doping semiconductor nanocrystals. Nature 436, 91–94 (2005). 16001066 10.1038/nature03832

[b29] Buonsanti R. & Milliron D. J. Chemistry of doped colloidal nanocrystals. Chem. Mater. 25, 1305–1317 (2013).

[b30] Shim M. & Guyot-Sionnest P. n-type colloidal semiconductor nanocrystals. Nature 407, 981–983 (2000). 11069172 10.1038/35039577

[b31] Haase M., Weller H. & Henglein A. Photochemistry and radiation chemistry of colloidal semiconductors. 23. Electron storage on zinc oxide particles and size quantization. J. Phys. Chem. 92, 482–487 (1988).

[b32] Liu W. K., Whitaker K. M., Kittilstved K. R. & Gamelin D. R. Stable photogenerated carriers in magnetic semiconductor nanocrystals. J. Am. Chem. Soc. 128, 3910–3911 (2006). 16551089 10.1021/ja060488p

[b33] Garcia G. . Dynamically modulating the surface plasmon resonance of doped semiconductor nanocrystals. Nano Lett. 11, 4415–4420 (2011). 21859093 10.1021/nl202597n

