# Supplementary material for: Direct observation of narrow mid-infrared plasmon linewidths of single metal oxide nanocrystals
Source: Nat Commun. 2016 May 13;7:11583. doi: 10.1038/ncomms11583 (PMC4869256; doi:10.1038/ncomms11583)
Supplement: Supplementary Information — Supplementary Figures 1-9, Supplementary Tables 1-3, Supplementary Notes 1-9, Supplementary Methods and Supplementary References. [file ncomms11583-s1.pdf]

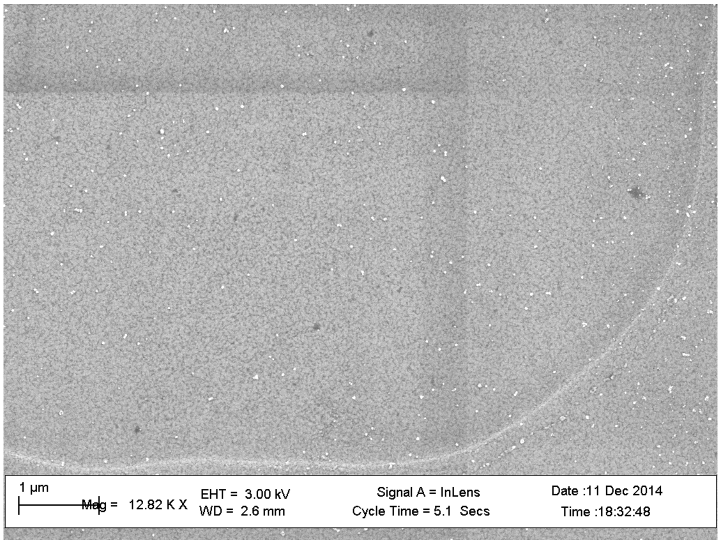

Supplementary Figure 1: Marked SINS substrates. SEM image depicts 100 nm pitch depression in substrate that was used to define an area in which to make measurements.

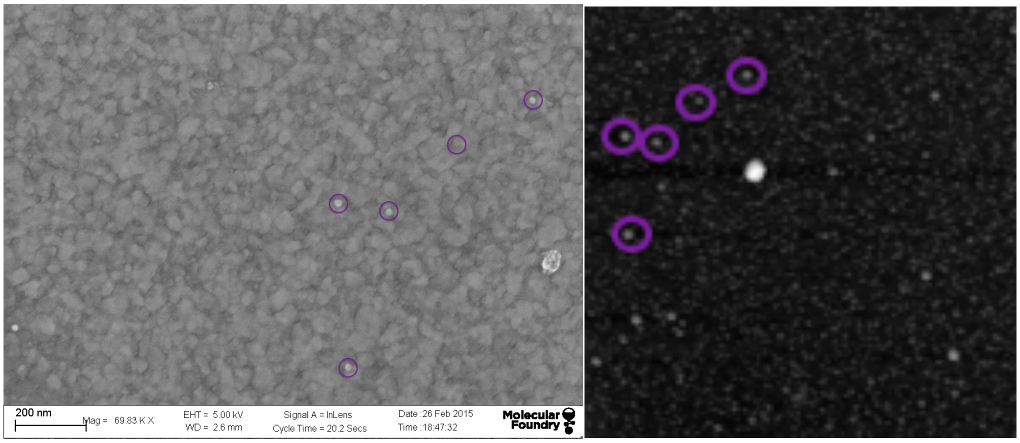

Supplementary Figure 2: SEM to AFM correlation. SEM image (left) and AFM image (right, full scale 1.9 μm) showing an example of image correlation verification. Purple circles are used to indicate the same arrangement of nanocrystals found around the large clump of nanocrystals apparent in both images.

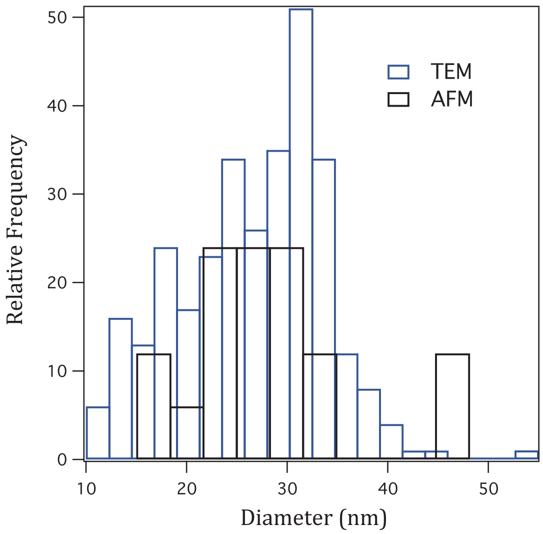

Supplementary Figure 3: Nanocrystal size distributions collected by AFM and TEM. Histograms of AZO nanocrystal diameters, measured with the AFM of the SINS instrument as well as from TEM analysis of the sample show these two techniques yield similar size distributions.

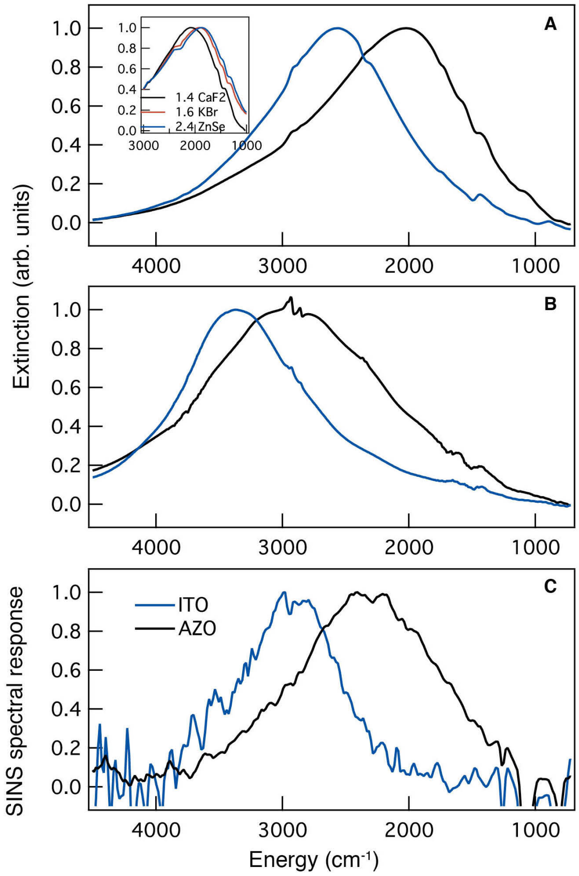

Supplementary Figure 4: Substrate dependence of ensemble LSPR spectra. Spectral shift of ensemble measurements of both AZO and ITO compared by A)

Transmission FTIR on KBr, as in Fig. 2G (comparison to various dielectric substrates in inset, refractive indices labeled), B) polarized specular reflectance FTIR on a gold substrate and C) pseudo-ensembles created from the sum of SINS scans. Inset shows the AZO LSPR shifting as it is deposited on different dielectric substrates.

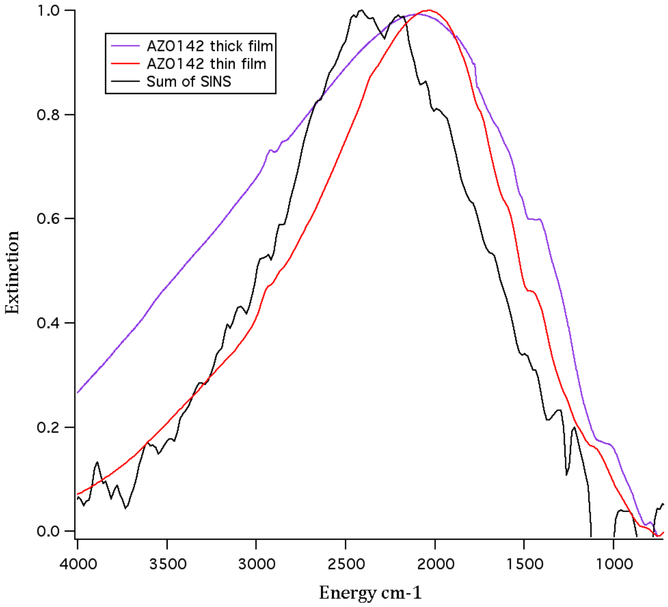

Supplementary Figure 5: Nanocrystal film thickness dependence of ensemble LSPR spectra. The SINS pseudo-ensemble for AZO is compared to an ensemble spectrum measured on an ultra-thin film (as in the main text Figure 2G) and to the spectrum of a thick film (roughly 4 nanocrystals thick) of the same nanocrystals.

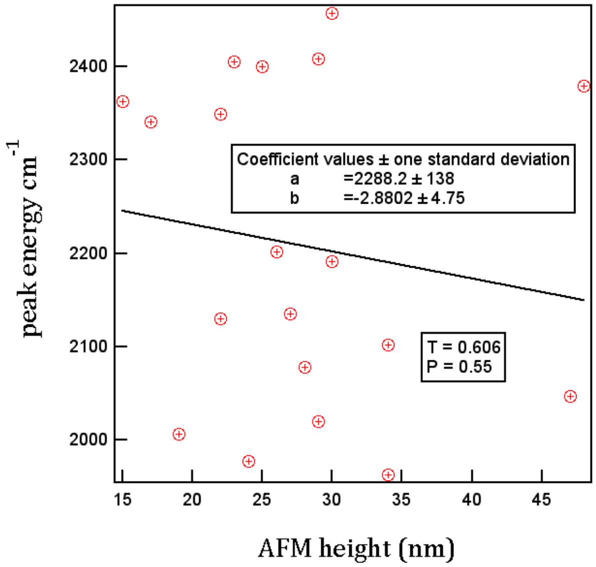

Supplementary Figure 6: Correlation check of nanocrystal size to LSPR peak energy. Single AZO nanocrystal LSPR peak energy and height from AFM correlation plot indicates no significant correlation.

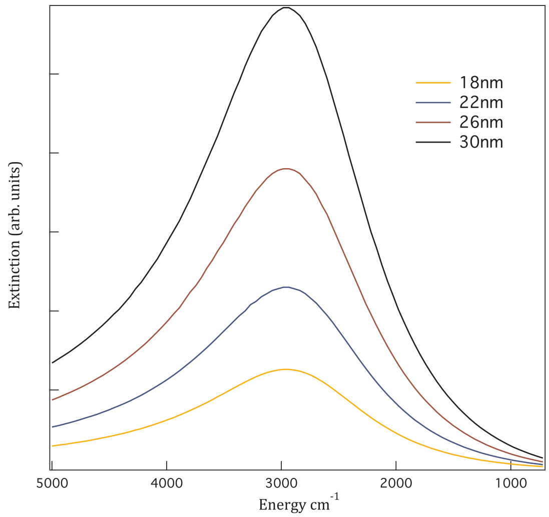

Supplementary Figure 7: Simulated size dependence of LSPR. Simulated mid-IR LSPR (3000 cm^{-1}) spectra are displayed of nanocrystals of sizes from 18-30 nm. No size dependence is expected by Mie theory except for absolute extinction intensity.

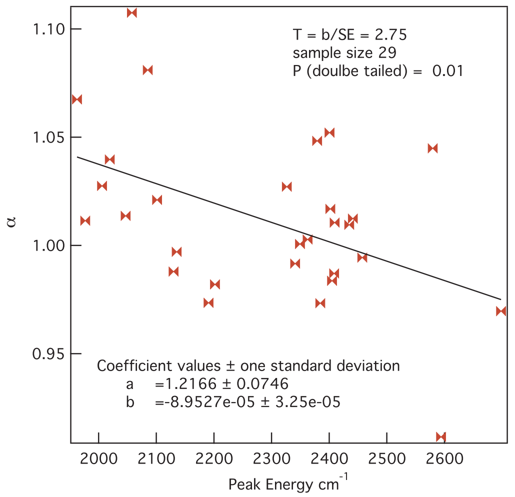

Supplementary Figure 8: Verification of the correlation between peak asymmetry and peak energy. Illustration of how statistical correlation was demonstrated between these two LSPR features.

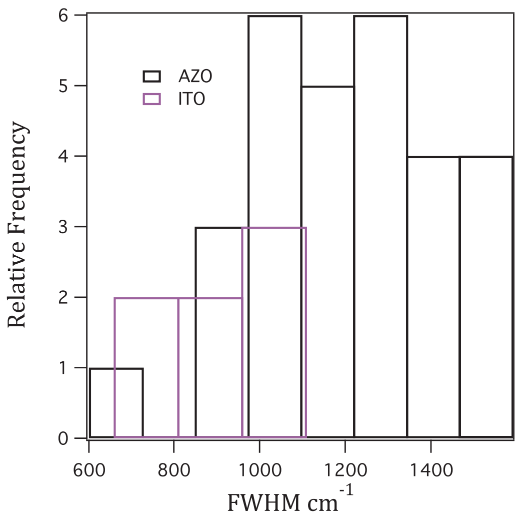

Supplementary Figure 9: Peak widths observed of single nanocrystal LSPR spectra. Histograms of the FWHM values observed in SINS of both ITO and AZO samples.

Supplementary Table 1: Table of collected SINS spectral values

	FWHM (cm-1)	Peak Energy (cm-1)	α
Range ITO	659-1105	2600-3016	0.86-0.93
Range AZO	602-1588	1962-2698	0.91-1.11
Median ITO	905	2941	0.88
Median AZO	1208	2349	1.01
Psuedo ensemble ITO	1033	2991	1
Psuedo ensemble AZO	1357	2330	0.99
FTIR ITO (KBr)	1141	2561	1.02
FTIR AZO (KBr)	1328	2036	1.07

Supplementary Table 2: Drude fit carrier concentration values

Variable Carrier concentration		
S.No.	N_c (cm^{-3})	Γ (cm^{-1})
Blue	2.17E+20	898.69
Black	2.90E+20	898.69
Red	3.63E+20	898.69

Supplementary Table 3: Drude fit frequency dependent damping values

Frequency Dependent Damping					
S.No.	N_c (cm^{-3})	Γ_L (cm^{-1})	Γ_H (cm^{-1})	Γ_X (cm^{-1})	Γ_W (cm^{-1})
Blue	2.17e+20	898.69	898.69	2200	400
Black	2.17e+20	1470	510	2200	400
Red	2.17e+20	2200	0	2200	400

Supplementary Note 1: SEM correlations to AFM scans
Each sample prepared for single particle measurements was produced by spin coating a dilute suspension of nanocrystals in 50/50 v%/v% hexane/octane onto marked gold coated substrates. These markings were several micron square indentations as depicted in Supplementary Figure 1, which provided a region to perform an AFM scan within that could easily be identified in the SEM. Then nanocrystal arrangements could be correlated using their relative coordinates within these areas in order to verify single nanocrystals had been measured by SINS (Supplementary Figure 2).

Through this type of image correlation it was revealed that in the ITO sample, roughly half of the nanocrystals measured were in fact not single nanocrystals, but instead two or more nanocrystals together. After eliminating all such clusters or dimers from the sample set, the final sample size for ITO nanocrystals used in the analysis was seven, all confirmed to be single nanocrystals. For the AZO sample, groupings of nanocrystals were far less common and, due to the larger size of these nanocrystals, single particles were more easily distinguished, even from the AFM topography data. Subsets of the AZO nanocrystals were verified as singles to establish confidence in interpreting AFM images, and on this basis nearly all AZO nanocrystals measured were included in the data set. Three spectra with extremely large linewidth (exceeding 1800 cm^{-1}) were excluded as likely clusters. In all, 29 AZO nanocrystals were included in the analysis.

Supplementary Note 2: Synchrotron infrared nanospectroscopy (SINS) details
Beamline 5.4 of the Advanced Light Source (ALS) at Lawrence Berkeley National Laboratory provides IR synchrotron radiation for the near field instrument used in these measurements[1]. This SINS set up consists of an asymmetric Michelson interferometer in which a KBr beamsplitter sends half of the light to a specially modified AFM (Innova, Bruker) and the other half to a modified commercial FTIR spectrometer (Nicolet 6700, Thermo-Scientific). Light reflected from the moving mirror of the spectrometer and the scattered light from the AFM tip are recombined on the beamsplitter and directed to a liquid nitrogen cooled mercury cadmium telluride (MCT) detector. A 700–5,000-cm^{-1} spectral range is obtained in this instrument through interferometric heterodyne detection.

Through methods first developed for dispersive Fourier transform spectroscopy[2], free-induction decay s-SNOM [3], and nano-FTIR[4,5]; signal can be processed from an asymmetric interferometer to obtain spectral information from small samples. SINS can be used to obtain the unique scattering coefficient for the region beneath which a platinum silicide (Nanosensors, PTSi–NCH) AFM tip is oscillating around ω_t=300 kHz. Because the near-field scattered signal has a non-linear distance dependence, detection at higher harmonics of ω_t with a lock-in amplifier (Zurich Instruments) discriminates the near-field signal from

background scattered light off the tip shaft, sample and substrate [6,7]. The value of the signal intensity comes from several contributions; the spectral profile of the ALS $E_{ALS}(\omega)$, the instrument's background absorption and scattering of frequencies in the IR, $R(\omega)$, and the scattering function of the region underneath the 20 nm-radius tip's profile $\sigma(\omega)$. Raw interferrograms are apodized (Hamming) and then Fourier transformed to obtain the complex spectral response $S_n=\sigma_n(\omega)R(\omega)E_{ALS}(\omega)$ where n is the nth harmonic, in this case the 2nd harmonic. By obtaining the quotient of sample signal to a background taken from a nearby place on the gold surface to minimize changes in $R(\omega)$ the scattering functions can be obtained. $S_{samp}/S_{bkg} = \sigma_{samp}(\omega)/\sigma_{Au}(\omega)$.

The scattering function can be processed in order to obtain the real and imaginary spectra, which relate to the complex dielectric function of the material. The imaginary component correlates well with the absorption of the material[21] and this component is reported in the spectra shown. For ITO nanocrystals only, the spectra were smoothed using a Savitzky-Golay(7) algorithm due to a lower signal to noise ratio ascribed to their much smaller volume.

In order to isolate the scattering function of the material it must be deconvoluted from that of the Au background[8]. There is a change in scattering intensity from the AFM tip when it is above a gold surface versus a nanocrystal. This is associated with a decrease in the near field strength due to the dielectric function of a more insulating material beneath the tip instead of a metal. Additionally, the increased distance of the tip from the substrate leads to a decrease signal. This weaker electric field leads to less excitation of non-spectrally flat background contributors to $\sigma_{Au}(\omega)$, causing some frequency dependence of background signal that cannot be separated from the spectral signature of the sample. Typically, the tips utilized in this experiment are processed and shipped on poly(dimethylsiloxane) (PDMS, gel-pak). This polymer off gasses during shipment and storage and it is well established that AFM tips shipped on PDMS maintain a layer of the polymer on them unless extensive washing procedures are performed[23]. The negative spectrum of PDMS is apparent in the reported spectra (peaks at 802, 1095, and 1265 cm^{-1}).

Additionally a broad background appears in all scans that must be subtracted due to the frequency dependence of scattering from the tip. Over the range of 700-4000 cm^{-1} this background can be treated linearly. This is corrected by fitting a linear baseline to regions at energies above and below the plasmon peak, roughly evenly spaced, more than 3 standard deviations away from the center of the peak in order to be in a region where even asymmetric peaks will have returned to zero to within the noise of the instrument. Then this linear function is interpolated over the full spectrum and subtracted. Repeat scans performed on the same nanocrystal each had individual backgrounds taken from a different regions of clean gold from nearby. Linear backgrounds varied from scan to scan

due to the area dependent background near field strength, so the same regions of the spectra were fit for each linear baseline interpolation for consistency.

Supplementary Note 3: AFM to TEM size distribution comparison
AFM heights of AZO nanocrystals selected for SINS analysis were compared to the TEM-derived size distribution (Supplementary Figure 3). Comparison of diameters measured show that representative distributions of nanocrystal sizes were measured for the SINS dataset. Although the smallest nanocrystals found by TEM (< 15 nm) were not among those measured by SINS and a slight selection bias for larger nanocrystals may be present, the SINS sample set is overall a reasonable reflection of the sample as a whole.

Supplementary Note 4: Substrate dielectric effects on LSPR spectra
A single dispersion of AZO nanocrystals was made that would form a submonolayer film with roughly 50% coverage when dropcast. Films were then deposited onto KBr, CaF_2, and ZnSe FTIR substrates and measured in transmission mode in a Bruker Vertex 70 FTIR (inset Supplementary Figure 4). The peak maximum shifted with refractive index of the substrate as expected for the influence of dielectric environment on nanocrystal LSPR spectra.

Gold, when used as a substrate for ensemble measurements induces a substantial blue shift. Measurements performed on gold were collected in specular reflection geometry with an incident angle of 80 degrees from normal. A polarizer optic was used to ensure that light was p-polarized with respect to the gold surface. Nanocrystal films used for these ensemble measurements were similar to the samples used for SINS, although the higher loading of nanocrystals required to achieve acceptable single to noise ratio meant that they were not as well isolated as in the SINS measurements (Supplementary Figure 4). Still, films of nanocrystals that were less thick than a monolayer were used in order to minimize broadening effects that occur in thicker films (more than a monolayer) that can also obscure peak position. As seen in Supplementary Figure 5, film thickness has a substantial impact on the FWHM and peak shape of ensemble spectra. Although no far field measurement could be a perfect comparison to the single nanocrystal spectra measured by SINS, these comparisons are considered the most relevant to evaluating SINS as a technique for recording single nanocrystal spectral characteristics.

Supplementary Note 5: Statistical correlation analyses
To establish a statistically significant correlation it must be demonstrated that the slope of the relationship between two variables is non-zero. Because there is always a probability that randomly distributed points could create a line of non-zero slope generally a P test is performed which takes a specific slope to standard deviation ratio (T value) and a given number of data points and calculates the probability that T can be obtained through random point

distribution. A low probability, generally $P<0.05$ satisfies the condition that there is very little chance that random points could create the given data set and the correlation is accepted as significant. For example from Figure 3A in the main text the ratio of the slope and standard deviations is used to find a P value of 0.01 for these data indicating significant correlation (see Supplementary Figure 8).

On the other hand, our analysis indicates that the correlation between particle size and the energy of the LSPR peak is not statistically significantly different from zero (Supplementary Figure 6). In the case of these data the standard deviation is larger than the slope yielding a quite high P value of 0.55, meaning it is more than 50% likely that this distribution of points could have occurred randomly.

This result may seem surprising when compared to the often observed size dependence of LSPR energy reported in single particle measurements collected with dark field scattering of metal nanocrystals. However, size dependence does not affect particles of sizes within the quasi-static approximation (diameters less than $\lambda_{LSPR}/20$). While for particles with LSPR in the visible particles cross this threshold around the size limit of 15-45 nm which is often the range of sizes that are measured, mid-IR LSPR with wavelengths an order of magnitude larger do not cross this threshold until sizes larger than 100 nm are reached. To illustrate this we simulated the expected LSPR size dependence for particle sizes relevant to our study (Supplementary figure 7).

Supplementary Note 6: Mathematical expression for the dependence of nanocrystal optical properties on electron concentration and damping parameters

Free electron concentration and frequency dependent (or independent) damping greatly influence the dielectric properties of a material. In our work we have modeled the dielectric function using a modified Drude-Lorentz model given by the following equation:

$$\varepsilon_p(\omega) = \varepsilon_\infty - \frac{{\omega_p}^2}{\omega^2 + i\omega\Gamma(\omega)} \quad (1)$$

Where ε_p is the frequency-dependent dielectric function, ε_∞ is the high frequency dielectric constant (here, 3.7), $\Gamma(\omega)$ is the frequency dependent damping function and ω_p is the bulk plasma frequency. The bulk plasma frequency is given by

$$\omega_p = \sqrt{\frac{N_c e^2}{\varepsilon_0 m_{eff}}} \quad (2)$$

Where N_c is free carrier concentration, e is electron charge, ε_0 is vaccum permittivity and m_{eff} is the effective mass (here, $0.6\,m_e$).

The frequency dependent damping function is an empirical function used to express the change in the dominant scattering mechanism with frequency due to

ionized impurity scattering (at low frequency) to grain boundary scattering (at high frequency). This damping behavior, which influences peak asymmetry, can be expressed using the following equation[9]:

$$\Gamma(\omega) = \Gamma_L - \frac{(\Gamma_L - \Gamma_H)}{\pi}\left[\arctan\left(\frac{\omega - \Omega_\Gamma}{\Gamma_\omega}\right) + \frac{\pi}{2}\right] \quad (3)$$

Where $\Gamma(\omega)$ is the frequency dependent damping, Γ_L is the low frequency damping constant, Γ_H is the high frequency damping constant, Ω_Γ is the crossover frequency, and Γ_ω is the crossover width.

The difference between Γ_L and Γ_H determines whether the material has frequency dependent or independent scattering.

Using the dielectric function obtained from equation 1, absorbance for a spherical particle is calculated using Mie theory under the assumption of subwavelength particle dimensions (the quasi-static approximation), so that the absorption is given as[10]:

$$C_{abs}(\omega) = 3Vk(\varepsilon_H)^{1/2}\,\mathrm{Im}\left\{\frac{\varepsilon_p(\omega) - \varepsilon_H}{\varepsilon_p(\omega) + 2\varepsilon_H}\right\} \quad (4)$$

Where k is the wavevector, V the nanocrystal volume, ε_H the host dielectric constant, and $\varepsilon_p(\omega)$ the material dielectric function.

The parameters used in the simulation of variable electron concentration are tabulated in Supplementary Table 2 and for frequency dependent damping in the Supplementary Table 3.

Supplementary Note 7: Single particle elemental analysis

In order to verify that dopant concentration variations were responsible for the observed variations in LSPR energies we performed energy dispersive spectroscopy. These measurements were performed on a FEI Titan Transmission Electron Microscope at the National Center for Electron Microscopy, Molecular Foundry, Lawrence Berkeley National Lab. The microscope was operated in STEM mode at 200 kV accelerating voltage. The STEM probe had a convergence semi-angle, α, of 10 mrad and a beam current of 700 pA. The STEM images were acquired using a Fischione high-angle annular dark-field (HAADF) detector. The EDS detector is an FEI Super-X Quad windowless detector based on silicon drift technology. The data were analyzed using the Bruker Esprit EDS analysis package, which has been calibrated against mineral standards for quantitative accuracy. Each map is a summation of 100 frames. K-edges of Zn, Al, O, Si, and Cu were fit by a least squares method after subtracting the Bremsstrahlung background. The Si peak was deconvoluted from the Al peak and the Cu peak was deconvoluted from the Zn peak. Peaks were integrated and atomic percentages were calculated based on a Cliff-Lorimer

factor method, which assumes that the sample is thin enough for single scattering.

EDS results reveal that for a sample of 14 AZO nanocrystals, the average concentration of Al was 1.6%, and had a range of 1.3% to 2% with a standard deviation of 0.2%. By comparison, the expected standard deviation in dopant distribution for 25 nm nanocrystals with an average dopant concentration of 1.5% is only 0.007%, based on Poissonian statistics. This value was obtained by first calculating the expected number of dopants within a 25nm nanocrystal at 1.5% doping, then using the relationship of σ=sqrt(mean) for this type of distribution. Finally, this σ value was converted back into percent using the expected total number of atoms in a 25 nm nanocrystal (42 zinc atoms/nm^3 in ZnO). This is clearly much less than the observed distribution. Although the sample size for both the single crystal EDS and SINS studies is relatively small, due to the challenging nature of these experimental techniques, comparing the range of measured dopant concentrations is informative to demonstrate the self-consistency of our EDS values, SINS spectra, and modeling efforts. The total range of Al content measured by EDS, about 44% relative to the mean, is comparable to the 50% range in carrier concentration needed to explain the variation in single particle LSPR energies measured by SINS. This range of LSPR energy is 32% relative to the maximum of the pseudo ensemble peak, in accordance with the LSPR energy being proportional to the square root of carrier concentration. These values are satisfyingly consistent, especially considering that not all dopants yield carriers, and that LSPR energy can also shift slightly due to changes in damping.

Supplementary Note 8: Dephasing time estimation

LSPR dephasing lifetimes can be estimated from homogeneous linewidths using the lifetime limited dephasing relationship.

$$\Gamma_{tot} = \frac{2\hbar}{T_2} \quad (5)$$

Although the possibility of multimodal contribution and increased temperature from the absorption of IR light is ever present, the observed minimum linewidths can still be considered as establishing an estimated lower bound for the lifetime of the LSPR.

Supplementary Note 9: Distributions in FWHM and discussion of linewidth and damping

FWHM values over a wide range were measured for the single nanocrystals (more broadly distributed in AZO than in ITO, Supplementary Figure 9). As discussed in the main text, the contribution that dopants make to FWHM (causing asymmetric peaks with $\alpha < 1$) is certainly one form of electron damping in doped oxide LSPR. Even the narrowest LSPR observed (Figure 4 in main text) have $\alpha <$ 1, which indicates that the variations in FWHM observed are likely not dopant

related but instead have another origin. Further evidence is found in the lack of the correlation between FWHM and energy, while these parameters would be expected to be correlated if the variation in FWHM was primarily due to dopants.

In light of dopants not being the primary contributor to FWHM variation one can consider the other commonly discussed damping pathways described in the literature: intrinsic bulk damping, electron surface scattering, interfacial scattering, ionized impurity scattering, and radiation damping[11].

$$\Gamma_{tot} = \gamma_{bulk} + \Gamma_{e-surf} + \Gamma_{\text{int}} + \Gamma_{ion} + \Gamma_{rad}$$

Nanocrystals on the order of 20 nm have been reported as having a 1/1000 scattering/absorption ratio making scattering and radiation damping negligible in these systems especially when one considers that the relationship of scattering efficiency to wavelength (λ^{-4}) would make scattering even less likely in the IR[12]. Also, with an electron mean free path in AZO of only a few nanometers[13] surface scattering should play a minimal role. Since it is unlikely that intrinsic bulk damping is changing dramatically from nanocrystal to nanocrystal it seems that variations in FWHM are derived primarily from variations of nanocrystal shape or different interactions between the nanocrystal and the substrate. Considering the obvious shape heterogeneity in our samples, we consider this factor to be the likely dominant source of heterogeneity in FWHM, though variations in nanocrystal-substrate interactions could only be ruled out completely were possible to measure single nanocrystal LSPR in a more homogenous dielectric environment, such as in solution phase.

Supplementary Methods: Synthesis details

Materials: Indium (III) acetylacetonate ($In(acac)_3$, 99.99%), tin (IV) bis(acetylacetonate) dichloride ($Sn(acac)_2Cl_2$, 98%), 1,2-Hexadecanediol (HDDIOL 99%), zinc (II) stearate ($Zn(st)_2$), aluminum (III) acetylacetonate ($Al(acac)_3$, 99.99%), oleic acid (technical grade), and octadecene (ODE technical grade) were purchased from Sigma Aldrich and used as received. Oleylamine (OLAM, 80-90%) was purchased from Acros and used as received.

ITO nanocrystals were synthesized based on a modification of a literature procedure[14]. All synthetic steps were carried out under nitrogen atmosphere using standard Schlenk line techniques and magnetic stirring.

To synthesize ITO nanocrystal seeds with 1% tin, 287 mg $In(acac)_3$ (0.7 mmol), 2.8 mg $Sn(acac)_2Cl_2$ (0.007 mmol), and 2.3 g OLAM (8.6 mmol) were mixed in a 25 mL 3-neck round bottom flask. The solution was heated to 110°C for 10 minutes under nitrogen, and then heated to 250°C for 2 hours. After the synthesis, the reaction mixture was precipitated with ethanol, centrifuged, and redispersed in hexane. After three additional washing cycles with ethanol and hexane, the nanocrystal seeds were dispersed in hexane at a concentration of 189 mg/mL.

ITO nanocrystals were synthesized by combining 180 μL of the concentrated ITO seed solution in hexane, 231.4 mg $In(acac)_3$ (0.56 mmol), 2.61 mg $Sn(acac)_2Cl_2$ (0.006 mmol), and 2.3 g OLAM in a 25 mL 3-neck round bottom flask. The solution was heated to 110 C for 10 minutes under nitrogen, and then heated to 250°C for 5 hours. After the synthesis, the nanocrystals were purified using the same methods as for the ITO seeds before finally dispersing the purified nanocrystals in hexane.

AZO nanocrystals were prepared[15] from two solutions: 10 mmol of 1,2-HDDIOL in 11mL of ODE, as well as 1mmol of $Zn(st)_2$, 0.3mmol $Al(acac)_3$, 3 mmol Oleic Acid and 4mL of ODE. Both solutions were heated for 1 hour at 140°C under nitrogen. Then the first solution was heated to 260°C. Once this temperature was reached, the second solution was rapidly injected into the first, then the temperature was held at 240°C for 5 hours. Nanocrystals were washed using the same procedure as above and then redispersed in 50/50 v/v hexane/octane.

Supplementary References:

1 Bechtel, H. A. Muller, E. A. Olmon, R. L. Martin, M. C. Raschke, M. B. Ultrabroadband infrared nanospectroscopic imaging. *Proc. Natl. Acad. Sci. U.S.A.* **111**, 7191–7196 (2014).
2 Birch, J.R. Dispersive fourier-transform spectroscopy. *Mikrochim Acta* **3**, 105–122 (1987).
3 Xu, X.G. Rang, M. Craig, I.M. Raschke, M.B. Pushing the sample-size limit of infrared vibrational nanospectroscopy: From monolayer toward single molecule sensitivity. *J. Phys. Chem.* Lett **3,** 1836–1841(2012).
4 Huth, F. *et al.* Nano-FTIR absorption spectroscopy of molecular fingerprints at 20 nm spatial resolution. *Nano Lett.* **12** 3973–3978 (2012).
5 Huth, F. Schnell, M. Wittborn, J. Ocelic, N. Hillenbrand, R. Infrared-spectroscopic nanoimaging with a thermal source. *Nat. Mater.* **10,** 352–356 (2011).
6 Hillenbrand, R. Keilmann, F. Complex optical constants on a subwavelength scale. *Phys. Rev. Lett.* **85,** 3029–3032 (2000).
7 Hillenbrand, R. Knoll, B. Keilmann, F. Pure optical contrast in scattering-type scanning near-field microscopy. *J. Microsc*. **202,** 77–83 (2001).
8 Govyadinov, A.A. Amenabar, I. Huth, F. Carney, P.S. Hillenbrand, R. Quantitative measurement of local infrared absorption and dielectric function with tip-enhanced near-field microscopy. *J. Phys. Chem. Lett.* **4,** 1526–1531 (2013).
9 Mergel, D. Qiao, Z. Dielectric modelling of optical spectra of thin In_2O_3 : Sn films. *J Phys D Appl Phys* **35,** 794–801 (2002).
10 Bohren, C.F. Huffman, D.R. Absorption and Scattering of Light by Small Particles (Wiley, New York 1998) chap. 5.
11 Olson, J. *et al.* Optical characterization of single plasmonic nanoparticles. *Chem. Soc. Rev.* **44**, 40–57 (2015).
12 Lo, Y. S. *et al.* Organic and inorganic contamination on commercial AFM cantilevers. *Langmuir* **15**, 6522–6526 (1999).
13 Pei, Z.L. *et al.*, Transparent conductive ZnO:Al thin films deposited on flexible substrates prepared by direct current magnetron sputtering. *Thin Solid Films* **497,** 20-23 (2006).
14 Choi, S.-I. Nam, K. M. Park, B. K. Seo, W. S. Park, J. T. Preparation and Optical Properties of Colloidal, Monodisperse, and Highly Crystalline ITO Nanoparticles. *Chem. Mater.* **20**, 2609–2611 (2008).

15 Buonsanti, R. Llordes, A. Aloni, S. Helms, B. A. Milliron, D. J. Tunable infrared absorption and visible transparency of colloidal aluminum-doped zinc oxide nanocrystals. *Nano Lett.* **11**, 4706–4710 (2011).
